# When Do Annuity-Based Payments Help to Address the Affordability Challenge of Funding Advanced Therapies? Insights from a Budget Impact Simulation

**DOI:** 10.3390/jmahp14020023

**Published:** 2026-04-20

**Authors:** Walter Van Dyck, Sissel Michelsen, David Veredas, Isabelle Huys, Jeroen Luyten, Steven Simoens

**Affiliations:** 1 Healthcare Management Centre, Vlerick Business School, 9000 Ghent, Belgium; sissel.michelsen@hotmail.be (S.M.); david.veredas@vlerick.com (D.V.); 2 Department of Pharmaceutical and Pharmacological Sciences, KU Leuven, 3000 Leuven, Belgium; isabelle.huys@kuleuven.be (I.H.); steven.simoens@kuleuven.be (S.S.); 3 Faculty of Economics and Business Administration, Ghent University, 9000 Ghent, Belgium; 4 Leuven Institute for Healthcare Policy, KU Leuven, 3000 Leuven, Belgium; jeroen.luyten@kuleuven.be

**Keywords:** advanced therapy, affordability, spread payment, annuity, upfront payment, budget impact

## Abstract

Spreading payments over time by means of annuities has been proposed as a means to address the affordability challenge of funding very expensive advanced therapies, especially within managed entry agreements. This study aims to examine when annuities (in contrast with a single upfront payment) offer a viable solution for both healthcare payers and manufacturers to fund one-time advanced therapies. We put forward four conditions under which annuity-based payments can be considered an acceptable payment strategy: (1) excessive budget impact, (2) cost equivalence with upfront payment, (3) compensation for financial risk and (4) a limited annuity period. We develop an exploratory model that simulates how the budget impact of annuity-based payments for advanced therapies meets these conditions across several economic and epidemiological scenarios. Given our model parameter values, results suggest that annuity-based payments are most suitable when the initial patient volume (prevalence) significantly exceeds annual new cases (incidence), and when the financial risk premium for the annuity-based payment scheme does not exceed the social discount rate. While further refinement of the model is needed, this study demonstrates that annuity-based payments can only help control the annual budget need when the focus is on a high-prevalence disease, and the therapy is financed through health impact bonds issued by a governmental payer. This arrangement ensures a low-risk premium, which is typically only available to public payers.

## 1. Introduction

Worldwide, healthcare systems are struggling with the competing objectives of controlling healthcare costs and providing maximal health benefits for their populations. This is certainly the case when it comes to funding advanced therapies. These products tend to be very expensive and offer the potential to cure patients, even though their long-term effectiveness is uncertain [1,2]. In particular, advanced therapies are often considered to be ‘cost-effective but unaffordable’ [3,4,5]. Several studies have shown the potential of advanced therapies to be cost-effective or even cost-saving due to their clinical benefit in terms of generating health and quality of life [6]. However, their high upfront prices may cause an immediate affordability challenge, limiting their market access across Europe and the US [7]. Therefore, if healthcare systems wish to fund advanced therapies, the budget impact problem must be solved.

Annuity-based payments (ABPs) in combination with managed entry agreements have been proposed as a solution to increase patient access to advanced therapies [8,9,10]. This means that the manufacturer and the healthcare payer agree on the number and the size of ABP (and potentially on other financial- and/or outcome-based conditions) during reimbursement negotiations. Even though ABPs, in combination with managed entry agreements, seem to facilitate market access to advanced therapies from a theoretical perspective, their practical implementation is fraught with difficulties, and the way in which they are implemented varies between countries [11,12,13,14].

The available literature seems to suggest that ABPs are a universal funding solution for all advanced therapies and has not yet explored the effect of spreading payments on the healthcare system [15,16,17]. This line of thinking contrasts with the findings from a literature review and a series of expert discussions organized by the US Institute for Clinical and Economic Review, which has described a ‘sweet spot’ where the use of annuities would be most suitable. This is when the therapy is indicated for a large enough population to warrant concern about the budget impact, and the treatment is curative with a high certainty on the durability of effect [18,19]. Conversely, this suggests that ABPs are not always an appropriate payment strategy for advanced therapies.

The aim of this study is to examine when ABPs offer a viable solution for use in managed entry agreements for advanced therapies. To this effect, we propose a number of conditions under which ABPs can be acceptable to both the healthcare payer and the manufacturer. Then, we construct an exploratory budget impact simulation model to describe the drug costs of providing an advanced therapy to a patient population growing over 25 years, either funded through an upfront lump-sum payment or an ABP. Varying relevant circumstances with regard to the epidemiology of the disease, economic circumstances and time preferences that drive patient volumes and the financial feasibility of a payment scheme, we investigate whether the conditions are satisfied under which ABPs can offer a solution to fund advanced therapies.

## 2. Materials and Methods

### 2.1. Literature Review

A set of conditions under which ABPs provide a funding solution for advanced therapies was developed by the authors based on a scoping review of the literature discussing payment options for advanced therapies and of financial/economic concepts. A search was conducted in PubMed and in Google Scholar using search terms including ‘single payment’, ‘upfront payment’, ‘spread payment’, ‘annuity’, ‘annuity-based payment’, ‘advanced therapy’, ‘funding’, ‘affordability’ and ‘budget impact’. These search terms were used individually or in combinations. The literature search was limited to documents written in English, French, Dutch or German. No time restriction was applied, given that the literature on funding advanced therapies is recent. Additional documents were identified by searching the bibliographies of relevant studies using the snowball sampling method.

### 2.2. Budget Impact Simulation Model

We developed a budget impact simulation model in Microsoft Excel to track drug costs for patients over a 25-year generation T, the main parameters of which are summarized in Table 1. Our model accounts only for drug purchasing costs and does not consider the potential offsetting disease-related cost savings generated by the advanced therapy, nor the durability of the therapy’s impact on health outcomes. The model focuses on an advanced therapy administered once. Model input data are summarized in Table 1. The values assigned to model parameters are used for illustrative purposes and reflect the characteristics of existing advanced therapies (or diseases for which these therapies are used) or are based on assumptions. They are varied over ranges considered to be realistic to the problem at hand.

Patient demographics and disease epidemiology influence volumes over time and subsequently the healthcare payer budget. Prevalence (*prev*) equals incidence (*inc*) times the duration of the disease. The total number of patients to be treated with the advanced therapy consists of an initial prevalent population (or backlog number of patients) and a yearly incidence-based population, evolving at a factor *g* over time, the latter caused by changing demographics. A prevalence-dominant disease population (*prev*/*inc* » 1) exhibits a high backlog of patients *prev* prior to treatment at t = 0 (many patients waiting for the treatment to arrive), typically when the duration equals life expectancy. Hemophilia B, a chronic genetic disease with an incidence of 1 in 25,000 male births and a life expectancy of 75 years, is an example. In our model, we use Leber’s Congenital Amaurosis (LCA) treated by gene therapy Luxturna^®^, which only treats LCA caused by the RPE65 gene mutation, featuring an *inc* of 1/750,000 newborns and a life expectancy of 70 years, which brings the disease *prev* to 1/10,700 [20].

In contrast, an incidence-dominant disease population (*prev*/*inc* ≤ 1) is characterized by a continuously high number of new patients *inc* and a lower number of patients waiting to be treated, e.g., through premature mortality. As an example, Kymriah^®^ and Yescarta^®^ for diffuse large B-cell lymphoma (DLBCL) have no backlog of existing patients because patients pass away quickly. Next, the budget needs to serve a flow of new patients entering a relapsed state every year. In our model, we took an *inc* of 2.5/1,000,000 patients and, with an average disease duration of 0.5 years without durable care, this leads to a *prev* that is half of the *inc* [21]. The *inc* growth factor *g* was varied up to 2%.

**Table 1:** Input data for budget impact simulation model.

Input Parameter	Leber LCA	DLBCL
Time horizon	25 years	
Maximum Yearly Budget	€250 million	€10 million
Patient prevalence	1/10,700	1.25/100,000
Patient incidence	1/750,000 newborns	2.5/100,000
Price of advanced therapy	€1 million	€350,000
Number of annuity-based payments	5, 10, and 15 years	
Social discount rate	3%	
Corporate Bond Rate	8–10%	
Health Impact Bond Rate	1–3%	

Note: Values based on authors’ assumptions or illustrative of existing advanced therapies [20,22].

Informed by Belgian list prices of a range of one-time advanced therapies (including Strimvelis^®^, Kymriah^®^, Yescarta^®^, Luxturna^®^, Zynteglo^®^ and Zolgensma^®^) [22], we used an average upfront cost of €1 million for the prevalence-dominant case (LCA) and €350,000 for the incidence-dominant case (DLBCL).

Paying for an advanced therapy following an annuity-based scheme of duration *n* and annuities *A*, the budget impact *BI_A_* in period *t* equals the portion *m* of the *prev* served in period *t*, which is *prev*/*m* × *A* and an *inc* portion such that

(1) $${BI}_{A,t}=A*\left(\frac{prev}{m}+\sum_{T=t}^{t-(n-1)}{inc}_{t}{\left(1+g\right)}^{t}\right)$$

At the end of each year, we calculate the budget impact for the entire patient population, both for upfront payments and for five-, ten-, or fifteen-year ABP schemes. Given that these costs occur in the future, they must be inflation-adjusted and discounted at a social rate of time preference, *r*, typically 3%.

Furthermore, ABPs consist of the upfront price *P_U_* spread over the number of annuity years *n*, plus a financial bond rate *i*, which varies between 3% when the national payer issues the bond, or up to 10% (or more when issued by a biotechnology company), when the innovator company issues the bond on the capital market.

The simulation output compares the socially discounted budget impact of upfront lump-sum payments with that of an ABP over 25 years. We use this model to investigate whether and how a managed entry agreement fixing *n*, *i*, and a negotiated upfront price *P_U_* can be reached between a healthcare payer and manufacturer whilst meeting our four conditions.

Our budget impact simulation model sheds light on what we call the feasibility space, or the range of epidemiological and economic characteristics that make ABPs a suitable method for funding advanced therapies. In the epidemiology-determined feasibility space for ABPs, we vary patient prevalence and incidence rates for two scenarios: LCA versus DBLCL. In the economy-determined feasibility space for ABPs, we map the extent to which health-bond-financed ABPs can mitigate budget impact requirements over the 25-year horizon.

## 3. Results

### 3.1. Literature Review

In the literature, the study of barriers to ABP implementation has predominantly been organizational, focusing on regulatory, data collection, and governance hurdles [8,23,24,25]. However, to assess the applicability of ABPs in managed entry agreements for advanced therapies, the perspective of the contractual relationship between the manufacturer and the healthcare payer should be taken into account. This is because a managed entry agreement will be agreed upon only if both parties believe that mutual gains from the exchange have been sufficiently realized.

Informed by the literature, we developed a set of four basic conditions, equally applicable to both private and public healthcare payer contexts, that have to be met for ABPs to be mutually acceptable as a payment solution for advanced therapies: excessive budget impact (condition 1), cost equivalence (condition 2), compensation for financial risk (condition 3) and minimal spread over time (condition 4) [26,27,28]. These conditions are explained in detail below.

#### 3.1.1. Condition 1: Upfront Payment Must Exceed a Maximum Yearly Budget Impact

Advanced therapies should receive funding if they are cost-effective (or cost-saving). In reality, however, healthcare systems do not set priorities solely on the basis of a cost-effectiveness comparison among interventions. Instead, they use a combination of criteria to make funding decisions [29]. One of these is the projected yearly budget impact of a new treatment and the system’s long-term sustainability.

Therefore, *Condition 1* to qualify for an ABP states that the upfront drug cost multiplied by the number of patients affected by the advanced therapy has to surpass the budget impact threshold, while the total annual payments under an ABP scheme have to be below it. So, the payer will have to define a maximum net yearly budget impact, *BI_max,_* for the patient population. This *BI_max_* can be defined in absolute terms or relative to the current standard of care, depending on the budgetary approach preferred by the decision maker. For instance, *BI_max_* can be set equal to the budget impact of the standard of care plus a fixed amount (e.g., as has been the case in England with the net budget impact test of £20 million per year for the patient population in the National Health Service [8] or in the Netherlands where a budget impact higher than €2.5 million per year qualifies drugs for conditional financing [30,31]). In our simulation, the annual budget impact is adjusted over *T* using a social discount rate, *r,* of 3%.

#### 3.1.2. Condition 2: There Must Be Cost-Equivalence Between Upfront Payment and ABP

If ABPs were a vehicle to increase the prices of advanced therapies even further, this would not be considered an acceptable solution to healthcare payers. Paying with annuities cannot, all things considered, be more costly to the healthcare system than paying upfront.

Cost-equivalence can be interpreted in two ways. Typically, one judges total costs over the total generation time horizon *T* in which the consequences of advanced therapies are meaningful. Given the epidemiological characteristics of the disease targeted by the advanced therapy, the socially discounted net present value (NPV) of the budget impact over the total generation *T* of ABP *BI_A_* should be no greater than that of the upfront payment *BI_U_*. Given that future payments need to be discounted at a rate *r*, *Condition 2a* is stated in the following equation [32]:

(2) $$\sum_{t=0}^{T}\frac{{BI}_{A,t}}{{\left(1+r\right)}^{t}}\leq \sum_{t=0}^{T}\frac{{BI}_{U,t}}{{\left(1+r\right)}^{t}}$$

In a stricter interpretation of cost-equivalence, the healthcare payer might not want the ABP budget impact *BI_A_* to exceed the upfront payment-based budget impact *BI_U_* in any finite future time period following the annuity period, since the payer would not like to trade off present gains for permanent future losses. We address this by comparing the maximum annual healthcare system budget impact (*BI_U, Max_*) under upfront payment with the maximum yearly BI (*BI_A, Max_*) under ABP. A *BI_A, Max_*/*BI_U, Max_* ratio of no more than 1 is preferred. We call this *Condition 2b*.

#### 3.1.3. Condition 3: Manufacturers Must Receive a High-Enough Risk Premium for ABP

For manufacturers to accept ABP, the total upfront payment must match the mortgage-like annuity *A* repayment schedule over a designated number of annuity years (*n*). This arrangement carries an opportunity cost of capital for the manufacturer (represented by the financial risk premium rate *i*. Hence, *Condition 3* stipulates that manufacturers are able to accept ABP [32] if

(3) $$P_{U}={NPV}_{A}=\sum_{t=1}^{n}\frac{A_{t}}{{\left(1+i\right)}^{n}}$$

and A is the annuity due at the beginning of each payment period and is expressed as a function of the principal P [32] as

(4) $$A=\frac{i*P}{1-\left(1+i^{-n}\right)}*\frac{1}{1+i}$$

Typically, financing facilities for these mortgage-like payment schemes can be obtained on the health impact bond market by the manufacturer or healthcare payer. Clearly, obtaining financial facilities on public governmental markets will come at a lower interest rate *i* than if obtained by the manufacturer as a corporate bond on private capital markets [29,33].

As an example, for an advanced therapy priced at a one-time upfront €1 million per patient, with a 3% government bond issued on capital markets paid with annuities over five years (*n* = 5), €211,995 is due at the beginning of each year. When a corporate bond is issued by the manufacturer on private capital markets, the financial risk premium could rise to *i* = 10%, with a corresponding annuity due of €239,816.

#### 3.1.4. Condition 4: The Number of Annuities Must Be Minimized

Both the healthcare payer and manufacturer will want the number of annuities *n* to be minimized, for reasons of intergenerational equity for payers (to avoid burdening future generations, as checked for in *Condition 2*) and financial risk for manufacturers.

Given a payer-determined *BI_max_* (*Condition 1*) and a financial market-obtainable health bond rate (*Condition 3*), the maximum number of annuity periods, *nmax*, is limited by the measured durability of the effect of the one-time advanced therapy [33]. If, for example, the efficacy of an advanced therapy becomes highly uncertain after approximately 30 months, this discourages the use of annuities longer than two years. Perpetuities are not advised, given the burden posed on future healthcare budgets and the risk of limiting the innovation of rapidly improving advanced therapy technology by manufacturers. This makes the use of longer-term, high-*n* ABP schemes less effective for incidence-dominant diseases with very short durations than for prevalent-dominant diseases.

### 3.2. Budget Impact Simulation Model

In our experimental design, we simulated annuity-based payments for both prevalence-dominant and incidence-dominant disease indications, using 5-, 10-, and 15-year bond payment schemes, and assessed whether the conditions specified above were met for the potential implementation of ABP.

#### 3.2.1. ABP Potential for Prevalence-Dominant Disease Indications

**Figure 1:**
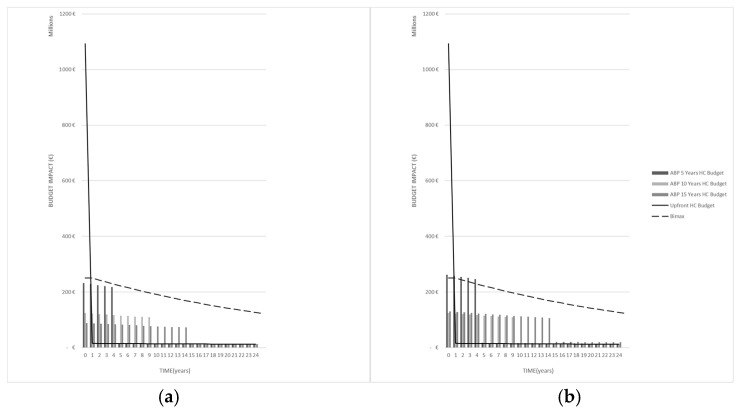
Maximum annuity payer budget impact (discounted) over generation *T* for *prev*-dominant disease domain at (**a**) health impact coupon bond rate of *i* = 3% and (**b**) corporate bond rate *i* = 10% for 5-, 10-, and 15-year maturity.

A visual inspection of Figure 1 shows that, for the predominant disease indication listed in Table 1, *Condition 1* is satisfied for an ABP scheme across all three maturities when the health impact bond risk premium is 3% or lower. However, this is not the case for a 10% premium, especially when a 5-year corporate bond is considered.

Table 2 presents the simulation results for *Condition 2a* and *Condition 3* for the corporate and health impact bond schemes for *prev*-dominant disease indications, respectively, across their three bond maturities.

As can be verified in Table 2, ABP can be implemented for *prev*-dominant disease indications, making use of health impact bonds of 10- and 15-year maturities. Making use of 5-year maturity health impact bonds could be envisaged, but this would be only slightly acceptable (0.98) for the manufacturer. The use of a corporate bond would not be acceptable for the healthcare budget holder, unless it were a 15-year bond maturity, which would also be acceptable for the manufacturer. Finally, *Condition 4* is met, as bond maturities of up to 15 years are shorter than the envisaged lifetime of 75 years for LCA being treated by gene therapy Luxturna^®^.

#### 3.2.2. ABP Potential for Incidence-Dominant Disease Indications

**Figure 2:**
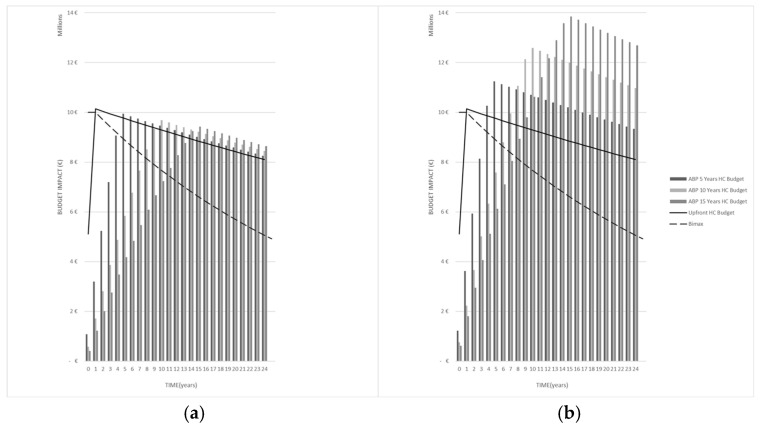
Maximum annuity payer budget impact (discounted) over generation *T* for *inc*-dominant disease domain at (**a**) health impact coupon bond rate of *i* = 3% and (**b**) corporate bond rate *i* = 10% for 5-, 10-, and 15-year maturity.

A visual inspection of Figure 2 shows that, for the *inc*-dominant disease indication listed in Table 1, *Condition 1*, being triggered by an upfront payment scheme surpassing *BI_Max_*_,_ is not satisfied for health impact bonds nor for corporate bond ABP schemes across all three bond maturities, while still surpassing *BI_Max_*.

Table 3 presents the simulation results for *Condition 2a* and *Condition 3* for the corporate and health impact bond schemes for *inc*-dominant disease indications, respectively, across their three bond maturities.

As shown in Table 3, ABP can be implemented for *inc*-dominant disease indications, using only 10-year health impact bonds. None of the other scenarios is acceptable to either the manufacturer or the healthcare budget holder. Finally, *Condition 4* is also not met, as a 10-year bond maturity is many times longer than the envisaged lifetime extension of a few years for DLBCL being treated by therapies like Kymriah^®^ or Yescarta^®^.

#### 3.2.3. Maximum Yearly Healthcare Budget Impact for Both Disease Indications

**Figure 3:**
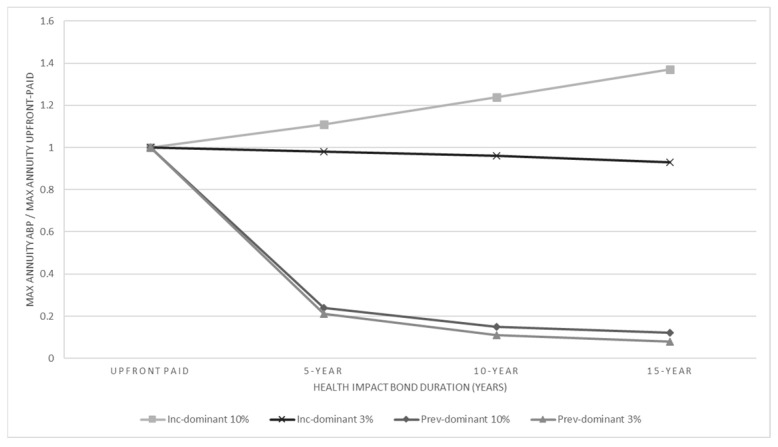
Relative maximum yearly healthcare budget need for *inc*- and *prev*-dominant disease domains at health impact bond and corporate bond rates.

When controlling *Condition 2b*, Figure 3 illustrates the ratio of *BI_U, Max_*_,_ the maximum annual budget impact of the healthcare system under upfront payment to *BI_A, Max_*, the maximum yearly budget impact under ABP over the entire timeframe *T*. A visual inspection of Figure 3 indicates that, for *prev*-dominant disease indications, irrespective of whether the financing is based on corporate bonds or health impact bonds, as well as the duration of the bonds, ABP yields a maximum yearly budget impact that is between 8% and 24% of the maximum yearly budget impact under upfront payment. So, for *prev*-dominant diseases, *Condition 2b* is positively met. In contrast, for *inc*-dominant disease indications, using ABP with corporate bonds yields a maximum annual budget impact that is between 11% and 37% higher than that achieved with an upfront payment. Additionally, *Condition 2b* is only slightly positively met when ABP is implemented using health impact bonds.

## 4. Discussion

This study has developed a budget impact simulation model to illustrate the circumstances under which ABPs for advanced therapies meet a set of conditions that make them a viable payment strategy for both healthcare payers and manufacturers. Taking into account that the circumstances and conditions considered were used for illustrative purposes and our model parameter values, the simulation model suggests that ABPs are preferred for disease indications where prevalence significantly exceeds incidence and when the financial risk premium equals the health impact bond rate, which is only obtainable by governmental institutions. Sensitivity analysis indicated that this rate cannot exceed 4% for ABPs to be preferred over upfront payment, regardless of therapy cost. It needs to be noted that epidemiological rates can be generally uncertain and vary widely between different countries, creating uncertainty regarding the treatable patient population. Therefore, the use of ABPs should be verified for the complete range of possible incidence and prevalence rates. Furthermore, it is unlikely that advanced therapies will attain a market uptake of 100% in light of their eligibility profile, the availability of alternative therapies, and patient/healthcare provider preferences. Limited market uptake can be considered in our budget impact simulation model by adjusting incidence and prevalence rates.

Our budget impact simulation model also indicated that, when a healthcare payer wishes to have an ABP, this will increase the price of an advanced therapy. A more critical payer not willing to trade off present gains with future losses will have to accept a therapy price that is corrected upwards by an increased financial risk premium.

Finally, although our study focused on ABPs as a potential approach to address the affordability challenge posed by advanced therapies, other innovative payment approaches exist. These include the establishment of a specific fund in a country to pay for innovative therapies, public sector investment in R&D of advanced therapies in return for lower list prices, re-insurance, subscriptions, and various types of financial and outcome-based managed entry agreements [34,35,36]. Also, multi-stakeholder funding approaches involving manufacturers, physicians, non-profit organizations and researchers have been proposed [37].

### 4.1. Study Strengths and Limitations

Our study has contributed to the debate about the applicability of ABPs to advanced therapies by proposing a number of conditions that make ABPs acceptable to a healthcare payer and a manufacturer and by exploring how epidemiological and economic characteristics determine when ABPs for advanced therapies satisfy these conditions. However, our study is also subject to limitations. Our scoping review of the literature generated little information about the conditions under which ABPs are a suitable payment mechanism for advanced therapies, and we, therefore, developed our own set of conditions. We do not claim that these conditions are exhaustive, and healthcare payers and manufacturers may consider additional conditions to be relevant. Also, their exact specification may be adapted. Instead, they should be seen as one possible minimal set of conditions that need to be satisfied for ABPs to be a viable solution for funding advanced therapies. Furthermore, as exemplified when discussing *Condition 4* (cfr. supra), these conditions are not independent from each other. Hence, decision makers need to reflect on which trade-offs they are willing to make. For instance, if they prefer to reduce the number of annuities, they need to consider raising the maximum affordable budget impact *BI_max_*.

Although our simulation model examined the role of several key characteristics in the choice between upfront payment and ABPs, it requires further refinement and validation. For example, we adopted the simplifying assumption that all prevalent patients are treated in the first year. This can be considered an extreme case, and in reality, it is likely to take multiple years to treat the backlog of existing patients. A sensitivity analysis shows that our finding—that ABPs are preferred only for prevalent-disease indications—remains valid even when the prevalent patient population is distributed over the first five years after its introduction, as simulated by values of *m* ranging from 1 to 5. This study explored when ABPs are a viable solution for funding advanced therapies for various realistic values of model parameters, but more extensive sensitivity analyses are required to elucidate the impact of uncertainty.

Our budget impact simulation model focused on drug costs and did not include the impact of therapy on other disease-related costs (or savings). Also, the model did not consider patient complexity (which may even vary within the same diagnosis-related group [38]). It should be acknowledged that patient complexity may influence drug and other disease-related costs and, thus, affect the budget impact of advanced therapies and the choice between ABP and upfront payment.

### 4.2. Recommendations for Future Research

Future research could focus on developing a generalized mathematical model that allows us to determine the suitability of ABPs versus upfront payment for advanced therapies. Such a model can also be more flexible and allow, for example, for the size of annuities to vary over time rather than being constant. Specifically, if ABPs are implemented in the context of an outcome-based managed entry agreement, the budget impact simulation model should allow for the possibility that the ABP is reduced when the durability of therapy effectiveness turns out to be shorter than expected or when a patient treated with an advanced therapy dies prematurely. Also, the simulation model needs to reflect a real-world setting in which some patients may be lost to follow-up or switch to another health insurance company. Finally, we recommend testing the budget impact simulation model in diseases for which advanced therapies are in development.

## 5. Conclusions

Even though ABPs are widely proposed as a vehicle to make funding advanced therapies more affordable, our exploratory budget impact simulation model suggests that ABPs are not always preferred over upfront payment. The selection of ABPs for advanced therapies is determined by disease epidemiology and the financial instrument involved. This can include either expensive corporate bonds or lower-priced health impact bonds, with the latter being accessible only to government institutions, which present the lowest risk to investors.

## Figures and Tables

**Table 2:** Results for *Conditions 2a* and *3* for *prev*-dominant disease indications.

Bond Type	Bond Maturity	*Condition 2a*	*Condition 3*
Health impact bond (*i* = 3%)	5-year	0.98	0.98
	10-year	0.96	1.17
	15-year	0.94	1.21

Corporate bond (*i* = 10%)	5-year	1.11	1.00
	10-year	1.25	1.73
	15-year	0.94	2.01


Note: Highlighted cells indicate where *Conditions* for ABP are met positively.

**Table 3:** Results for *Conditions 2a* and *3* for *inc*-dominant disease indications.

Bond Type	Bond Maturity	*Condition 2a*	*Condition 3*
Health impact bond (*i* = 3%)	5-year	0.93	0.93
	10-year	0.84	1.29
	15-year	6.02	1.19

Corporate bond (*i* = 10%)	5-year	1.05	0.97
	10-year	1.09	3.27
	15-year	6.02	3.4


Note: Highlighted cells indicate where *Conditions* for ABP are met positively.

## Data Availability

The original contributions presented in this study are included in the article. Further inquiries can be directed to the corresponding author.

## Abbreviations

The following abbreviations are used in this manuscript:

A	Annuity amount
ABP	Annuity-based payments
BI	Budget impact
BI_A_	Budget impact of annuity payments
BI_max_	Maximum yearly budget impact
BI_U_	Budget impact of upfront payment
HC	Healthcare
i	Financial risk premium
Inc	Incidence
n	Number of annuities
n_max_	Maximum number of annuity periods
NPV	Net present value
Prev	Prevalence
P_U_	Upfront price
r	Social rate of time preference
T	Time horizon
